# Microbiota and healthy ageing: observational and nutritional intervention studies

**DOI:** 10.1111/1751-7915.12048

**Published:** 2013-03-26

**Authors:** Harald Brüssow

**Affiliations:** BioAnalytical Sciences, Food and Health Microbiology, Nestlé Research CenterLausanne, Switzerland

## Abstract

Hundred years ago Metchnikoff associated human health and particularly healthy ageing with a specific type of gut microbiota. Classical culture methods associated a decrease in bifidobacteria and an increase in enterobacteria with ageing. Modern molecular methods blurred this simple picture and documented a substantial inter-individual variability for the gut microbiome even when stratifying the elderly subjects according to health status. Nutritional interventions with resistant starch showed consistent gut microbiota changes across studies from different geographical areas and prebiotic supplementation induced a 10-fold increase in gut bifidobacteria. However, in the ELDERMET study, microbiota changes do not precede, but follow the changes in health status of elderly subjects possibly as a consequence of diet changes.

Microbiota and its impact on human health has become a topical area of microbiological research. However, health is a vast field as explored in a parallel minireview (Brüssow, submitted) and the role of the gut microbiota on human health has been discussed under many headings. In the present minireview, I will concentrate on one specific aspect. Our societies are ageing and if we want to avoid that our societies will suffer from an increasing burden of disease, we must also assure healthy ageing. This subject has not only a high societal priority, it has also a long tradition in microbiology.

## Healthy ageing

In fact, the first association between microbes and healthy ageing was made by one of the founding fathers of modern microbiology and immunology, namely Elie Metchnikoff from Kharkov/Ukraine. He shared the Nobel Prize for Medicine with Paul Ehrlich in 1908 and published in the same year an influential book ‘The Prolongation of Life’ (Metchnikoff, 1908). As nicely described in a book review from the *New York Times*^1908^ he developed in this book the idea that higher animals needed an increasingly complex intestine to struggle for existence. Unfortunately, the intestine became the breeding place for poisonous microbes. The ravages of these poisonous microbes are not only the cause of disease (Koch's germ theory of disease), but from the detrimental metabolism of some gut bacteria spring the debility of old age leading to earlier death. Metchnikoff distinguished two types of metabolism for gut bacteria: proteolytic bacteria lead to putrefaction with noxious metabolites as waste products and saccharolytic bacteria lead to fermentation with beneficial metabolic end-products like lactic acid. He suggested combating putrefaction in the gut by hygiene, diet and biologicals. He recommended the consumption of boiled food and drinks since microbes enter the digestive tract in vast numbers with raw food. He called for appropriate mastication (quick eating leaving food lumps in the intestine which become then heavily colonized by gut microbes) and particularly he made an argument for milk fermented by lactic acid bacteria as food. The fermentative metabolism of lactic acid bacteria would counterbalance putrefaction by the noxious gut bacteria and their toxic effect on our tissues. He backed this theory by the observation that population showing traditionally high yoghurt consumption also showed increased longevity.

This idea was picked up in Japan and developed there into an industrial product. The Japanese microbiologist Shirota isolated the first probiotic bacterium in 1930 and developed this *Lactobacillus casei* strain into a dairy drink which was introduced on the market in 1935. Over the decades this sole product was used to develop a substantial nutrition and health business. Despite this early industrialization, over the following decades the microbiota of the human gut and its relationship to human health met only limited interest from applied and environmental microbiologists.

## Gut microbiota and ageing

A review on intestinal flora and ageing from the early 1990s reported that many factors in addition to ageing altered the composition of the gut microbiota (physiological state, drugs, disease, diet and stress) (Mitsuoka, 1992). The composition of the gut microbiota was known to affect physiological functions (e.g. digestion and absorption), drug efficacy, carcinogenesis, immune function and infection. Three groups of gut bacteria were distinguished. Symbiotic gut bacteria (*Bacteroidaceae, Eubacterium, Peptococcaceae, Bifidobacterium, Lactobacillus*), which are important for the maintenance of health via synthesis of vitamins, help in digestion, stimulation of immune function and their inhibition of pathogenic microbes. A second group comprised *Escherichia, Streptococcus* and *Veillonella*. Protein degradation by these bacteria leads to NH_3_, H_2_S, amines, phenols production and was linked – as originally proposed by Metchnikoff – to the production of toxic compounds and thereby possibly to ageing. The third group of gut bacteria were pathogens that induced infections. Research on the gut commensal *Escherichia coli* became a founding pillar of bacterial genetics and molecular microbiology. In contrast, until the mid-1990s, the gut microbiota in its ecological complexity was only investigated by a small research community using nearly exclusively culture-based methods. Since then a growing number of molecular methods were developed that allowed increasingly detailed, cheap and time-efficient approaches characterizing bacterial microbiota. Techniques like DGGE, T-RFLP, dot-blot hybridization, FISH, qPCR, phylogenetic microarrays and then particularly the sequencing of cloned 16S rRNA amplicons attracted many scientists to the field of gut microbiota (O'Toole and Claesson, 2010). In parallel, research into probiotic gut bacteria intensified (Woodmansey, 1993). With the current techniques of massive sequencing (454 pyrosequencing, Illumina technique) and the expanding arsenal of –omics techniques and major international research initiatives in the Human Microbiome Project, gut microbiota analyses and gut microbiota-human health studies reach top-ranking scientific journals (Clemente *et al*., 2012). This gut microbiome research is clearly exciting as an eye-opener for a previously unsuspected biological diversity in our body. It led to new ways of looking into human genetic diversity extending phenotype–genotype association studies from the few ten thousand human genes to the potentially million of genes encountered in our bacterial commensals. The field proposed widely quoted working hypotheses like the link between gut microbiome composition and human health: the most prominent being the association of the *Bacteroides/Firmicutes* ratio in gut microbiota with obesity. Overall, the field is, however, still in the state of botany in the 18th century when naturalists returned with countless new plants from expeditions into until then poorly explored parts of the world. A census was made and the diversity of plant life was described, but no theory was yet available to make sense out of this mind-bogging biological diversity. We are still in the census period of microbial diversity corresponding to the time of Linné in botany; instead of taxonomical methods we now use increasingly sophisticated statistical methods and hope to sort out associations which could lead us to new biological insight. The exciting progress of DNA sequence technologies and ‘–omics’ approaches are also a temptation to run more and more descriptive census approaches simply because they are technologically feasible. However, we also need the elaboration of biological hypotheses about the meaning of commensalism, host–microbe interaction and microbial diversity which makes explicit predictions that are tested with experiments and surveys. To stay with the botany analogy, the contribution of Russel Wallace is still missing in the field.

Despite an explosion in gut microbiome literature, data on the changes of the gut microbiota with old age are still limited. On the basis of studies using culture methods, the mid-1990 research situation could be summarized as followed: bifidobacteria diminished with age, while clostridia, lactobacilli, streptococci and enterobacteria increased with age and *Bacteroides* remained stable. Over the last 10 years, a handful of studies re-investigated the question. A study compared the faecal colony counts of seven young adults with that of four elderly subjects using culture methods and biochemical markers for species definition. The small number of investigated subjects reflects the laborious aspect of this approach. The authors found that *Bacteroides* species diversity increased and that bifidobacterial species diversity decreased in the elderly subjects (Hopkins and MacFarlane, 2002). In another study the same authors reported again a marked decrease in bifidobacteria, a slight decrease in lactobacilli, but no change in viable counts for the *Bacteroides*-*Prevotella* group in elderly compared with young adults. The bifidobacteria in the healthy elderly showed either high or negligible counts resulting in wide standard errors (Hopkins *et al*., 2001). In a follow-up study by the same Gut Microbiology Group at the University of Dundee/UK, 12 healthy young adults were compared with six healthy elderly subjects for faecal bacterial counts. The researchers observed a decrease in *Bacteroides* species diversity in elderly, but no overall decrease in bifidobacteria. However, the species composition of bifidobacteria changed with age: *B. angulatum* and *B. longum*, respectively, were the most common isolates in elderly and younger adults. Both *Bacteroides* and bifidobacteria showed reduced numbers in the faeces of the elderly subjects. Facultative anaerobes increased with age (Woodmansey *et al*., 2004). From these three papers published by the same research group investigating local subjects for gut microbiota composition, it is apparent that substantial inter-individual differences will make it difficult to arrive at general conclusions. The high variability of the gut microbiota composition was also documented by another group using a different analytical technique, namely oligonucleotide probes for fluorescence *in situ* hybridization of faecal samples coupled with flow cytometry. Overall, 85 young adults were compared with 145 healthy elderly subjects from four European countries. The French and Swedish study arms showed no age-related microbiota changes. Age effects were only seen in the German and the Italian study groups. However, when considering the predominant bacterial groups (*Eubacterium-Clostridium, Bacteroides-Prevotella*), the German elderly subjects showed an increase while the Italian elderly participants showed a decrease in faecal number for these bacteria when compared with the corresponding local younger subjects. Bifidobacteria were high only in Italian subjects, and that in both age groups (Mueller *et al*., 2006).

A French study using qPCR methodology to assess five bacterial genera documented a marked change in the ratio of *Firmicutes* to *Bacteroidetes* from 11 to 0.6 when comparing young with elderly adults. Elderly subjects exhibited high levels of *E. coli* and *Bacteroidetes* (Mariat *et al*., 2009). Another group investigated the gut microbiota in 21 centenarians, 22 seniors (mean age 73 years) and 20 adults younger than 40 years from Europe using the Human Intestinal Tract Chip (HITChip) and qPCR technology. No significant difference was seen between the young and elderly subjects. Even the microbiota composition of the centenarians was still similar to that of young adults: the centenarians showed only an increase in bacilli and in facultative anaerobes, but neither were a dominant faecal population. Interestingly, the centenarians showed an increase in pathogens (*Klebsiella pneumoniae*) and a slight, but significant decrease in *Faecalibacterium prausnitzii* (a symbiont with anti-inflammatory properties) when compared with seniors. This shift in microbiota composition was associated with an increased inflammatory status as determined by plasma levels of pro-inflammatory cytokines. The group coined the term ‘inflammageing’ for this chronic low-grade inflammatory status which they suggested as one possible driver of the ageing process (Biagi *et al*., 2010).

In 2007 the ELDERMET consortium was established to investigate the role of the intestinal microbiota in 161 Irish subjects older than 65 years as an agent and indicator of health. When the faecal microbiota of the study participants was investigated by pyrosequencing of 16S rRNA and compared with that of nine younger control subjects, the researchers observed an increase in *Bacteroidetes* from 41% to 57% of the sequences and a concomitant decrease in *Firmicutes* from 51% to 40% in younger and older subjects respectively. However, averaging hides a dramatic inter-individual variability in the composition of the elderly gut microbiota. The proportion of *Bacteroidetes* ranged from 3% to 92% in the different elderly subjects and that of *Firmicutes* varied from 7% to 94%. When plotted as a histogram, an essential smooth distribution for the *Bacteroidetes/Firmicutes* ratio was observed between the two extremes. The same characteristic was observed when the ratio of clusters IV and XIV was investigated within the *Clostridium* group. The wide distribution of the data was also seen for other bacterial groups, e.g. *Faecalibacterium* showed a steady distribution between 16% and < 1% prevalence. When the *Bacteroidetes* group was further differentiated, some differences between the young and elderly adults could be identified. Microbiota compositional differences were – as expected – greater between individuals than between individuals who were sampled twice (Claesson *et al*., 2011).

## Ageing and ailing

In the previous section, we have seen that ageing (except perhaps for extreme ageing) does not leave a consistent imprint on the gut microbiota. In fact, this observation is not surprising. When you have great inter-individual variability for a given parameter in a population as it is the case for gut microbiota, you need more precisely defined or quite drastic conditions to expect consistent changes in gut microbiota. Old age alone is apparently not a sufficiently strong disturbing factor to upset the gut microbiota composition, it might need addition events like antibiotic treatment (Bartosch *et al*., 2004; Woodmansey *et al*., 2004) or *Clostridium difficile* infection (Hopkins *et al*., 2001; Hopkins and MacFarlane, 2002) to get significant and consistent signal changes in elderly subjects. One study compared faecal microbiota in hospitalized elderly patients receiving and not receiving antibiotic treatment. Only a marginal reduction in total 16S rRNA gene copy numbers was seen in the antibiotic treated patients, but significant reductions in *Bifidobacterium, F. prausnitzii* and *Desulfovibrio* were induced by antibiotic treatment (Bartosch *et al*., 2004). Another study from the Dundee group showed a significant increase in total facultative anaerobe counts upon antibiotic treatment in elderly patients. The higher number of proteolytic bacteria in the stool of antibiotic-treated elderlies was also associated with an increased proteolytic species diversity particularly in fusobacteria, clostridia and propionibacteria (Woodmansey *et al*., 2004). The Dundee researchers compared also healthy elderlies with elderlies suffering from *Clostridium difficile*-associated diarrhoea (CDAD). CDAD patients showed reduced numbers of bifidobacteria and bacteroides, but increased numbers of enterobacteria (Hopkins *et al*., 2001). A follow-up study of the Dundee group comparing healthy with CDAD elderly subjects confirmed these conclusions (Hopkins and MacFarlane, 2002).

Since antibiotic treatment and *C. difficile* infection impact directly on the gut microbiota and are only indirectly related to the ageing process, these observations are unlikely to be informative for the microbiota changes with increasing age. Other criteria that are a more reliable measure for healthy and unhealthy ageing are needed for such an approach. The Dundee group addressed this hypothesis by comparing the faecal microbiota in 35 healthy elderly subjects living in the local community with that of 38 elderly patients who were hospitalized. They used real-time PCR techniques with group and species-specific primer sets. The *Bacteroides-Prevotella* group, *F. prausnitzii* and *Clostridium clostridiiformi* were all detected with lower numbers in stool of hospitalized patients when compared with community-living subjects. Bifidobacteria showed no significant differences between the groups. However, since the hospitalized subjects also showed a 10-fold lower total bacterial 16S rRNA gene count, the relative abundance of the different bacterial groups did not change with hospitalization (Bartosch *et al*., 2004). A Dutch study took a similar approach by assessing the faecal microbiota composition in 23 elderly subjects (median age 86 years) living in the same old age centre and receiving the same diet. The subjects were stratified according to the Groningen Frailty Indicator into 13 subjects with low frailty and 10 subjects with high frailty score. This time the total number of bacteria as assessed by hybridization with specific probes did not differ between both groups. Statistically significant differences were seen for the *Lactobacillus/Enterococcus* group (10-fold decrease in the high frailty group) and for *Enterobacteriaceae* (10-fold increase in high frailty group) (van Tongeren *et al*., 2005). Also the ELDERMET consortium stratified their data according to the health status of their elderly subjects into 83 community-dwelling, 20 outpatients, 15 short-term hospitalized subjects and 60 subjects with long-term residential care. The statistical analysis of the microbiota composition indicated a clear separation between community-dwelling subjects and long-stay home residents. The latter showed a higher proportion of *Bacteroidetes* while the former showed a higher proportion of *Firmicutes* and unclassified bacteria. *Lachnospiraceae* were a key species enriched in the community-living subjects (Claesson *et al*., 2012). Both groups differed also in faecal metabolome analysis. Acetate, butyrate, propionate were more abundant as metabolites in the community dwellers while glucose, glycine and lipids were at higher faecal levels in the long-term residents. The metabolome data were corroborated by a higher gene count for enzymes involved in the production of short-chain fatty acids (SCFA) in the community dwellers (Claesson *et al*., 2012). The data fit with current knowledge about bacterial metabolism and gut physiology. SCFA are known nutrients for the colonic enterocytes.

Within the ELDERMET study the community dwellers differed from the long-stay residents and rehabilitation subjects also for other parameters. The community dwellers showed a better score in a number of health/ frailty tests than the residents including the calf circumference, which is a good measure of muscle mass and an index for sarcopenia. The co-morbidity index was as expected higher in the long stay residents. Inflammation markers (IL-6 and IL-8) correlated with a specific gut microbiota type confirming data from Biagi and colleagues (2010). Notably, the effect of antibiotic treatment was eliminated as confounding factor from this study.

## Ageing, diet and microbiota

Diet correlated very strikingly with the health status in the ELDERMET study: 8% of the long-stay residents consumed a moderate to high-fat/low-fibre diet while 98% of the community dwellers displayed a low to moderate fat/high fibre diet. Interestingly, the diversity index of the faecal microbiota correlated positively with a low fat and high fibre content of the diet. The change in diet is partly the consequence of the food prepared at residence canteens. Due to a greater co-morbidity in residents compared with community dwellers, the diet change is also a consequence of their compromised physical situation (poor dentition decreasing mastication, dysphagia affecting swallowing, loss of salivation complicating lubrication of food, decreased physical activity resulting in reduced gut motility and thus constipation) – all resulting in a trend to offer ‘easier’ food items. Since food changes are expected to change the metabolism of gut bacteria and thereby also the microbiota composition, we are here confronted with a complicated epidemiological situation. Is the microbiota change observed in the long-stay residents a consequence of the diet change or a consequence of their deteriorating health or even one of the causal factors leading to health decline? An answer could be provided by a prospective study looking into an elderly cohort before and when a deterioration of the health status occurs. If the microbiota changes with decreasing health, but before diet changes occur, gut microbiota composition could be a driver for unhealthy ageing. If microbiota composition changes only secondarily after diet changes, microbiota changes are consequences of unhealthy ageing or even simpler of diet change. The ELDERMET study addressed this question by analysing the diet and microbiota type in residents according to residence time. Within 1 month after transfer to residence, all subjects had changed to the long stay-typical diet, but it took a year for the microbiota to get clearly separated from that of the community dwellers. Therefore microbiota changes do not precede, but follow the changes in health status of elderly subjects. In that study microbiota changes were the consequence of unhealthy ageing.

## Nutritional interventions: resistant starch

A recent study explored the effect of short-term feeding of a high-fat/low-fibre versus a low-fat/high-fibre diet given to 10 healthy adults for 10 days. Wide variation of microbiota composition was observed between the subjects at baseline and a 10-day intervention was not able to impose a diet-induced change in microbiota composition as determined by 16S rDNA sequencing. The authors concluded that changes in microbiota composition (expressed as alternative enterotype states) can only achieved by long-term dietary changes (Wu *et al*., 2011). Only few studies explored the impact of diet changes on the faecal microbiota using microbiota analysis. One study from UK investigated the effect of feeding diets rich in resistant starch (RS), rich in wheat bran and a low-carbohydrate/high-protein diet, respectively, to 14 obese human volunteers (Walker *et al*., 2011). 16S rDNA sequencing done with six subjects showed significant diet-induced changes for two species, namely *Eubacterium rectale* and *Ruminococcus bromii*, which accounted for 4% and 2% of all bacterial sequences. The inter-individual difference between the subjects was greater than the diet-induced intra-individual changes. A clearer picture emerged from qPCR analysis conducted with all 14 volunteers. It revealed significant increases of ruminococci in most volunteers after change to the diet rich in resistant starch. The change corresponded to a 10-fold increase in number such that *Ruminococcus* represented 25% of the total bacterial 16S rRNA. The change occurred within 3 days after diet change, but the microbiota returned as quickly to the baseline composition after stopping this diet. A few subjects showed a rise in *E. rectale* during this diet, but only two subjects showed increased counts for bifidobacteria. The other diets had no consistent effects.

Notably, this observation of a specific effect of resistant starch diet on *R. bromii* (and *E. rectale*) confirms reports from two different groups working with subjects from different continents (Abell *et al*., 2008; Martínez *et al*., 2010). When 46 healthy adults from Australia got resistant starch as a supplement to their diet at two different concentrations compared with a control diet in a cross-over design, diet-associated DGGE bands were detected in the stool. Half of the bands, which increased by the nutritional intervention were identified as *R. bromii*. A quantitative PCR showed that *R. bromii* increased from about 4.9% to 7.9% after supplementation with RS (Abell *et al*., 2008). In a US study 10 young adults received diets supplemented with native starch and two forms of RS in a cross-over format: RS2 was granular starch and RS4 was chemically modified starch. Compared with the control starch RS4 showed increases in bifidobacteria (mostly *Bifidobacterium adolescentis*), porphyromonadaceae, but decreases in ruminococcaceae. RS2 showed significant increases in *R. bromii* and *E. rectale*. The RS4-induced increase for *B. adolescentis* was substantial in a subset of the subjects (3–30% of the total bacteria with pyrosequencing) and thus also plainly visible on DGGE analysis (Martínez *et al*., 2010).

However, the beneficial effects of neither *R. bromii* nor *E. rectale* are established. However, when human stool samples were incubated with various nutrient substrates, the most abundant sequences recovered from starch particles were related to the cultured species *R. bromii, B. adolescentis, B. breve* and *E. rectale* (Leitch *et al*., 2007). *Eubacterium* belongs to the most abundant known butyrate-producing bacteria in human faeces (Hold *et al*., 2003). However, only bifidobacteria and lactobacilli have so far been clearly associated with health-promoting effects (Kleerebezem and Vaughan, 2009). What then is the evidence that the prevalence of faecal bifidobacteria could be boosted by nutritional interventions in elderly subjects?

## Nutritional interventions: prebiotics

A prebiotic has been defined as a non-digestible food ingredient that beneficially affects the host by selectively stimulating the growth and activity of one or a limited number of bacteria in the colon, and thus improves host health (Gibson and Roberfroid, 1995). This is a quite complex definition since it requires for a food ingredient that it should not be hydrolysed and absorbed in the upper part of the intestine, be a selective nutrient for few colonic bacteria, alter the colonic microbiota and induce beneficial health effects for the host. The non-digestible characteristics are associated with some complex carbohydrates and proteins. However, since only anaerobic saccharolytic, but not anaerobic proteolytic activity have been associated with health (anaerobic proteolysis producing in fact harmful compounds), only a handful of complex carbohydrates have qualified as prebiotics like fructooligosaccharides which include both short-chain oligofructose and longer-chain inulin (Hamilton-Miller, 2004). Pioneering work from the MRC Dunn Clinical Nutrition Center at Cambridge/UK showed that these compounds are *in vitro* selectively fermented by bifidobacteria at the expense of bacteroides, clostridia and coliforms (Wang and Gibson, 1993). In the colonic consortium fructooligosaccharides are metabolized into small chain fatty acids which are used by different host organs (butyrate by the colonic epithelium, propionate and lactate by the liver, acetate by the muscle). *Bifidobacteria* had immunostimulatory activities when given to elderly subjects as dietary supplement (Gill *et* *al*., 2001) to quote only one beneficial effect. Soon later, the Cambridge nutritionists showed that fructooligosaccharides (FOS) increased selectively the faecal counts for bifidobacteria from 10^8.8^ to 10^9.5^ and 10^9.2^ to 10^10.1^ cfu g^−1^ faeces, respectively, when oligofructose or inulin was fed to human volunteers (Gibson *et al*., 1995). German nutritionists achieved with inulin a very similar effect in elderly constipated subjects (Kleessen *et al*., 1997). Bifidobacteria titres increased in a dose-dependent way from 10^7.9^ to 10^8.8^ and 10^9.2^ cfu g^−1^ faeces when 20 and 40 g inulin per day was fed respectively. Inulin showed a slight beneficial laxative effect. Small chain FOS increased also bifidobacteria from 10^8.5^ to 10^9.2^ cfu g^−1^ stool and cholesterol excretion from stool in a study with healthy elderly volunteers from France (Bouhnik *et al*., 2007). However, both values returned to baseline 4 weeks after wash-out. Galactooligosaccharides (GOS) have been extensively used in paediatrics and a study with healthy 70-year-old subjects from UK showed likewise an increase in bifidobacteria from 10^9.1^ to 10^10^ cfu g^−1^ stool. GOS had no beneficial effect on cholesterol excretion with the stool, but stimulated natural killer cell activity, phagocytic activity and decreased the production of pro-inflammatory cytokines (interleukin-10 and tumour necrosis factor alpha) compared with placebo (Vulevic *et al*., 2008). A smaller *Bifidobacterium* increase was seen in 60-year-old healthy subjects from UK (10^8.9^ to 10^9.2^ cfu g^−1^ stool) which was significant only for women (Walton *et al*., 2012).

Gut microbiota is routinely determined by taking faecal microbiota as a proxy measure. Due to technical difficulties and ethical constraints, biopsies are rarely taken. One exception is the study from Langlands and colleagues (2004) where 60-year-old subjects scheduled for colonoscopy were supplemented for 2 weeks before the intervention with FOS and inulin or no supplement. Biopsy samples from the proximal and distal colon from both groups were cultivated for a panel of bacteria. In both compartments the prebiotic group showed increased bifidobacteria counts compared with the control group (10^6.4^ versus 10^5.2^ cfu g^−1^ mucosa). Lactobacilli and a specific butyrate-producing group from the *Clostridium* cluster (*Eubacterium*) showed likewise prebiotics-induced titre increases. Total anaerobe counts were unaffected and total aerobes showed a non-significant half-log decrease.

## Nutritional interventions: probiotics

In the early 1970s probiotics were described by Sperti as ‘organisms or substances which contribute to intestinal microbial balance’, only to be redefined in the late 1980s by Fuller as ‘a live food supplement which beneficially affects the host by improving its intestinal microbial balance’ (quoted from Gibson and Roberfroid, 1995). The possibilities and problems of probiotics was already highlighted in a study conducted 20 years ago with a *Bifidobacterium* strain which showed natural resistance to streptomycin and rifampicin and could thus be differentiated from the background endogenous bifidobacterial microbiota when given to human volunteers in milk fermented with this strain. One day after oral application, the strain reached a titre of 10^9^ cfu g^−1^ stool (Bouhnik *et al*., 1992). This titre was maintained as long as the oral supplement was given. Two days after stopping the *Bifidobacterium* feeding, the titres gradually decreased and became undetectable a week later. Overall, 30% of the orally applied bifidobacterium survived the gastrointestinal passage, but comparison with an inert control (*Bacillus* spores) suggested that no major amplification of the supplemented *Bifidobacterium* had occurred during *in vivo* passage. The gut passage was thus a rather passive transit. In healthy elderly subjects from New Zealand, feeding of milk containing various doses of *Bifidobacterium lactis* (known for its immunostimulatory effects; Gill *et* *al*., 2001) resulted in a dose-independent increase of faecal bifidobacteria counts from 10^9.3^ to 10^9.8^ cfu g^−1^ stool. Increases were also seen for faecal lactobacilli and enterococci, while enterobacteria were reduced when bifidobacteria were fed (Ahmed *et al*., 2007). No marked difference in faecal bifidobacterium concentrations were detected in Finnish elderly nursing home residents fed with a *B. longum*-containing drink compared with a placebo drink. However, when compared with the baseline value, the probiotics-receiving subjects showed a significant increase for *Bifidobacterium bifidum* and *B. breve* (Lahtinen *et al*., 2009).

The next logical step was combining the rationale of prebiotics and probiotics, by giving a mixture of probiotic bifidobacteria (*B. bifidum* and *B. lactis*) together with an inulin-based prebiotic to 18 healthy 70-year-old women (Bartosch *et al*., 2005). Total *Bifidobacterium* counts were increased in the symbiotic group (10^9.4^ cfu g^−1^ stool) when compared with the placebo group (10^8.3^ cfu g^−1^), but not when compared with the symbiotic pre-feeding period. The same observation was made for total lactobacilli counts. The results were confirmed by real-time PCR quantification of rRNA genes for all bifidobacteria. However, significant increases also with respect to the prefeeding period were detected when PCR was done with *B. bifidum*- and *B. lactis*-specific primers. Continuous excretion of the orally applied bifidobacteria was documented for all subjects into the first and for some subjects into the third post-feeding week suggesting limited probiotics persistence in elderly subjects getting synbiotics. Interestingly, supplementation with just inulin induced a significant increase in the endogenous *B. adolescentis* prevalence in the stools of human volunteers (Ramirez-Farias *et al*., 2009) while a symbiotic combination of inulin with only one bifidobacterium probiotic, namely *B. lactis* Bb-12 in young adults did not induce an increased total bifidobacteria faecal count during the feeding period when compared with the placebo group. Interestingly, total bifidobacterial counts were significantly increased in the post-feeding period, but this could not be attributed to the supplemented probiotic strain. The treatment had only small effects on the faecal clostridia and enterobacteria counts (Palaria *et al*., 2012).

## Outlook

Whether gut microbiota changes are statistically associated with ageing or are just a biomarker (i.e. a consequence) of or even a driver (i.e. a cause) for the ageing process cannot yet be decided from the available data. The current literature does not even allow assigning a typical and old age-specific composition for the gut microbiota of elderly subjects. Some trends were repetitively observed in old age (decrease of bifidobacteria, increase of enterobacteria), but they were far from universal. Major problems for assigning an old age microbiota profile are the high inter-individual variability and the clear impact of geography and diet on gut microbiota composition. Advanced age is probably not a sufficiently clear phenotype. Healthy and unhealthy ageing must certainly be differentiated to get to a more consistent picture. In addition, frailty and diseases come in many forms and each form has most likely its specific impact on the gut microbiota.

Like in the case of resistant starch studies it is important to get a consistent observation from different and independent studies to distil stable signals out of the relative ‘noise’ of the gut microbiota. Currently, we do not know whether the variability in the gut microbiota composition reflects clear biological rules which we are still unable to read for our limited understanding of the system or whether it represents intrinsic stochastic behaviour. However, sorting out a consistent microbiota picture is only the first step. The next step will be to decipher what changes are potentially detrimental and what re-established prior situations are beneficial. This will not be an easy task since systematic reviews of large epidemiological, nutritional and microbiological data sets will be needed to settle these questions. Finally, from a practical viewpoint knowing what gut microbiota is good and what is bad is not sufficient, we need to know how to get from the bad to the good composition. When limiting the complexity to simple models like gnotobiotic mice colonized with a community of 10 sequenced human gut bacteria, 60% of the microbiota changes following randomized perturbations of simple diets consisting of four defined ingredients can be explained with a statistical model (Faith *et al*., 2011). The data with the resistant starch dietary intervention are interesting for their consistent effect on specific gut bacteria in three different human populations. Since several microbiota-ageing studies reported decreases of bifidobacteria in old age and since bifidobacteria have an already fairly well-established role as probiotic organisms and since their prevalence can be influenced by specific prebiotic supplements, interventions increasing bifidobacteria and suppressing enterobacteria (another relative constant association with old age) should be conducted in elderly subjects to look for health improvements. Overall, it will be a long way to promote healthy ageing by nutritional interventions which target the gut microbiota. The task will be complex because such trials necessitate the collaboration of doctors, nutritionists, microbiologists and analytical chemists. However, it will be an exciting field for microbial biotechnology approaches and numerous are the people who would profit from such interventions if successful.
